# Specific tumor targeting and activation of Vγ9Vδ2 T cells by bi-specific nanobodies

**DOI:** 10.1186/2051-1426-2-S3-P127

**Published:** 2014-11-06

**Authors:** Anita Stam, Renée de Bruin, Rob Roovers, Paul van Bergen en Henegouwen, Henk Verheul, Tanja D de Gruijl, Hans van der Vliet

**Affiliations:** 1 Department of Medical Oncology, VU University Medical Center, Amsterdam, Amsterdam, Netherlands; 2 Division of Cell Biology, Department of Biology, Utrecht University, Utrecht, Utrecht, Netherlands

## 

Gamma delta T cells expressing the Vγ9Vδ2 T cell receptor (TCR) are the most predominant γδ-T cell subset in peripheral blood accounting for approximately 1-5 % of all T cells. Vγ9Vδ2 T cells recognize phosphoantigens (pAg) such as isopentenyl pyrophosphate (IPP), a naturally occurring pAg that can accumulate in tumor cells, resulting in activation, cytokine release and anti-tumor activity of Vγ9Vδ2 T. The use of Vγ9Vδ2 T cells in clinical trials, either via adoptive transfer of *ex vivo *expanded Vγ9Vδ2 T cells or through *in vivo *activation by aminobisphosphonates or synthetic pAg, has led to promising results. Anti-tumor responses were observed in some patients, but overall results lack consistency. This might be related to systemic activation of Vγ9Vδ2 T cells in these trials, not providing a specific trigger for these cells to accumulate at the tumor site.

In order to improve the efficacy of Vγ9Vδ2 T cell based immunotherapy, we focused on the design of a tumor-targeting construct that binds both the TCR of Vγ9Vδ2 T cells and the Epidermal Growth Factor Receptor (EGFR), which is over-expressed by many tumor types, including Vγ9Vδ2 T cell susceptible tumors like colon carcinoma and head and neck cancer. For this bi-specific construct an antagonistic anti-EGFR single domain antibody fragment (VHH or Nanobody) and an agonistic anti-Vγ9Vδ2 TCR VHH were identified, characterized and constructed into a bi-specific targeting molecule. Only when bound to both EGFR expressing tumor cells and Vγ9Vδ2 T cells, this bi-specific targeting molecule induced Vγ9Vδ2 T cell activation, release of IFN-γ and TNF-α as well as up-regulated expression of cytolytic molecules such as perforin-and granzyme B. Importantly, tumor targeted Vγ9Vδ2 T cells were able to efficiently lyse EGFR expressing tumor cells *in vitro*.

This study shows that bi-specific anti-Vγ9Vδ-T-anti-EGFR-nanobodies can specifically and efficiently lyse EGFR-expressing tumor cells and are promising candidates for cancer immunotherapy.

